# Faster skin wound healing predicts survival after myocardial infarction

**DOI:** 10.1152/ajpheart.00612.2021

**Published:** 2022-01-28

**Authors:** Mediha Becirovic-Agic, Upendra Chalise, Mira Jung, Jocelyn R. Rodriguez-Paar, Shelby R. Konfrst, Elizabeth R. Flynn, Jeffrey D. Salomon, Michael E. Hall, Merry L. Lindsey

**Affiliations:** ^1^University of Nebraska Medical Center, Omaha, Nebraska; ^2^Research Service, Nebraska-Western Iowa Health Care System, Omaha, Nebraska; ^3^Institute of Molecular and Translational Therapeutic Strategies, Hannover Medical School, Hannover, Germany; ^4^Department of Physiology and Biophysics, University of Mississippi Medical Center, Jackson, Mississippi; ^5^Division of Pediatric Critical Care, Department of Pediatrics, University of Nebraska Medical Center, Omaha, Nebraska; ^6^Department of Medicine, University of Mississippi Medical Center, Jackson, Mississippi

**Keywords:** heart failure, inflammation, myocardial infarction, remodeling

## Abstract

Both skin wound healing and the cardiac response to myocardial infarction (MI) progress through similar pathways involving inflammation, resolution, tissue repair, and scar formation. Due to the similarities, we hypothesized that the healing response to skin wounding would predict future response to MI. Mice were given a 3-mm skin wound using a disposable biopsy punch and the skin wound was imaged daily until closure. The same set of animals was given MI by permanent coronary artery ligation 28 days later and followed for 7 days. Cardiac physiology was measured by echocardiography at baseline and MI *days 3* and *7*. Animals that survived until *day 7* were grouped as survivors, and animals that died from MI were grouped as nonsurvivors. Survivors had faster skin wound healing than nonsurvivors. Faster skin wound healing predicted MI survival better than commonly used cardiac functional variables (e.g., infarct size, fractional shortening, and end diastolic dimension). *N*-glycoproteome profiling of MI *day 3* plasma revealed α_2_-macroglobulin and ELL-associated factor 1 as strong predictors of future MI death and progression to heart failure. A second cohort of MI mice validated these findings. To investigate the clinical relevance of α_2_-macroglobulin, we mapped the plasma glycoproteome in patients with MI 48 h after admission and in healthy controls. In patients, α_2_-macroglobulin was increased 48 h after MI. Apolipoprotein D, another plasma glycoprotein, detrimentally regulated both skin and cardiac wound healing in male but not female mice by promoting inflammation. Our results reveal that the skin is a mirror to the heart and common pathways link wound healing across organs.

**NEW & NOTEWORTHY** Faster skin wound healers had more efficient cardiac healing after myocardial infarction (MI). Two plasma proteins at *D3* MI, EAF1 and A2M, predicted MI death in 66% of cases. ApoD regulated both skin and cardiac wound healing in male mice by promoting inflammation. The skin was a mirror to the heart and common pathways linked wound healing across organs.

## INTRODUCTION

Myocardial infarction (MI) is the major underlying etiology for heart failure with reduced ejection fraction (1). Although the advent of reperfusion has dramatically improved 30-day survival rates, there remains a significant patient population who undergoes adverse remodeling and progresses to heart failure (2–5). Predicting how an individual patient will respond is an unmet clinical need.

Cardiac repair efficiency is a critical determinant of progression to heart failure following MI (6–8). Insufficient cardiac wound healing yields adverse remodeling that can progress to heart failure and premature death (6, 9). Skin wound healing progresses through a similar process as cardiac wound healing, including infiltration of inflammatory cells, inflammation resolution, and proliferation of fibroblasts to form scar tissue (6, 10–14). Due to the similarities between the two injury models, we hypothesized that the healing response to skin wounding could predict later response to MI. The wound healing process varies substantially even within the same mouse strain in part due to epigenetic mechanisms and germline mutations (15–19). Harnessing the variability in individual response, we evaluated skin wound healing and cardiac wound healing in serial in the same C57BL/6J mice, to understand how one process interrelates with the other.

High-throughput proteomics can give mechanistic insight into the process of wound healing as well as identify biomarkers for improved diagnostics (20). A limitation of current proteomic techniques is that albumin, a highly abundant plasma protein, limits the identification of less abundant plasma proteins (21, 22). Protocols and commercial kits for albumin depletion have been developed to solve this issue. However, these have proven to be problematic because a large number of plasma proteins bind to albumin, and therefore, are removed during the depletion. In addition, other proteins not bound to albumin are also depleted during the process, which further lowers the extraction yield (21, 23). Since albumin is not glycosylated and the majority of extracellular proteins are *N*-linked glycosylated, a glycoproteomic approach for plasma analysis is well suited to mitigate these limitations (21, 24). In this study, we mapped the plasma glycoproteome after skin wounding and MI in mice and after MI in humans to provide mechanistic insight into the process of skin and cardiac wound healing.

## METHODS

### Mice

Male and female, 3–6 mo old, C57BL/6J wild-type mice (Jackson Laboratory) were used in this study. The animals were housed in the same room with 12-h:12-h light/dark cycle, and free access to standard mouse chow and water. All animal procedures were conducted in accordance with the *Guide for the Care and Use of Laboratory Animals* (8th ed., 2011), and all protocols were approved by the Institutional Animal Care and Use Committee at the University of Mississippi Medical Center or the University of Nebraska Medical Center.

### Experimental Design

#### Cohort 1 (23 males, 19 females).

Mice were given a skin wound 28 days (*D−28*) before MI induction using a 3 mm disposable biopsy punch (Miltex). An image of the wound was taken every day until the wound was closed, and wound area pixels were measured using ImageJ (25). MI was induced by permanent left anterior coronary artery ligation according to previously described guidelines (2, 28). Buprenorphine (0.5 mg/kg sc) was given for analgesia before surgery. MI was confirmed at the time of surgery by presence of LV blanching and ST segment elevation on the electrocardiogram. MI was reconfirmed 72 h later by echocardiography.

Cardiac physiology was assessed by 2-D echocardiography using the Vevo 2100 for *cohort 1* and Vevo 3100 for *cohort 2* (VisualSonics, Toronto, ON, Canada) as previously described and in accordance with Guidelines for Measuring Cardiac Physiology in Mice (29, 30). Images were collected in B-mode for long axis view and in M-mode for short axis view. LV volumes and ejection fraction (EF) were calculated from the long axis view, and LV dimensions and fractional shortening (FS) were calculated from the short axis view.

**Figure 1.**
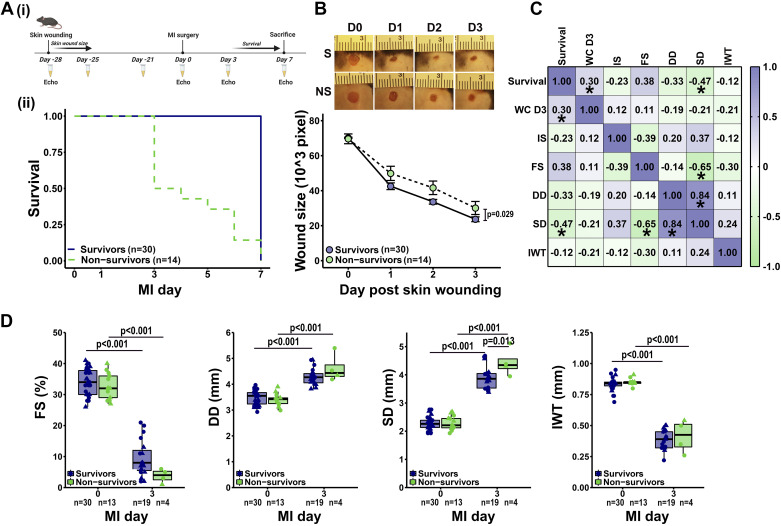
Skin wound closure rate predicted survival after myocardial infarction (MI). *A*,*i*: mice were given a 3-mm skin wound using a disposable biopsy punch. Skin wound was imaged every day until closure. The same cohort of mice were given MI 28 days later by permanent coronary artery ligation and followed for 7 days. Created with BioRender.com (accessed on 5 January 2022) (71). *A*,*ii*: animals that survived until MI *day 7* were grouped as survivors, whereas animals that died before *day 7* were grouped as nonsurvivors. *B*: survivors had faster skin wound healing compared with nonsurvivors. Data were analyzed using linear mixed effects model, and the *P* value shows the comparison between survivors and nonsurvivors. *C*: apart from end-systolic dimension (SD), wound closure rate [100% – (wound size/baseline wound size × 100)] at *day 3* (WC D3) predicted MI survival better than commonly used cardiac variables including infarct size (IS), MI day 3 fractional shortening (FS), end-diastolic dimension (DD), and infarct wall thickness (IWT). Data were analyzed using Pearson correlation analysis. *P < 0.05. *D*: as expected, MI reduced myocardial contractility (fractional shortening), induced dilation (diastolic and systolic dimension), and infarct wall thinning (wall thickness). Data were analyzed using linear mixed effects model with Tukey’s post hoc test. Circles represent males, and triangles represent females. The *n* represents the number of animals in each group.

Blood collection and echocardiography were performed at the days indicated in Fig. 1*Ai*. All surviving animals were euthanized at MI *day 7*. The 7-day follow-up time was selected because the kinetic rate of left ventricle (LV) remodeling (wall thinning and dilation) predominantly occurs over the first 7 days following MI (27).

#### Cohort 2 (10 males, 11 females).

Mice were given MI without previous skin wounding. Blood was collected at MI *days 3* and *7* for plasma analysis, and echocardiography at *days 0*, *3*, and *7*. All surviving animals were euthanized at MI *day 7*.

### Blood and Tissue Collection

Serial blood samples were collected from the submandibular vein into a heparinized tube. At the time of euthanasia, mice were injected with heparin (4 IU/g) and blood was collected from the jugular vein. Immediately after collection, the blood was centrifuged for 5 min, plasma separated, mixed with 10× protease inhibitors (9:1, Roche), and snap frozen in liquid nitrogen. Lungs were removed and weighed. The hearts were arrested in diastole by flushing the hearts with cardioplegic solution (69 mM NaCl, 12 mM NaHCO_3_, 11 mM glucose, 30 mM 2,3-butanedione monoxime, 10 mM EGTA, 1 µM nifedipine, 50 mM KCl). The LV was separated from the right ventricle, weighed, and cut into three pieces: base, middle, and apex. The LV pieces were incubated in warm (37°C) 1% 2,3,5-triphenyltetrazolium chloride (Sigma) for 5 min and imaged to determine LV infarct size (percentage of total LV area infarcted) using Adobe Photoshop (28, 31). For nonsurviving mice in *cohort 1*, infarct size was taken at the time of autopsy.

### Quantification of Glycoproteins

*N*-linked glycopeptides were isolated using solid phase extraction as previously described and quantified using liquid chromatography-tandem mass spectrometry (21, 24, 32, 33). Briefly, plasma proteins (20 µL plasma) were reduced, alkylated, and trypsin-digested to obtain peptides. Glycosylated peptides were oxidized and conjugated to a solid support. *N*-linked glycopeptides were released from the solid support using peptide-*N*-glycosidase F (PNGase F). The released peptides were quantified by label-free liquid chromatography-tandem mass spectrometry using Q Exactive (Thermo Fisher, Waltham, MA) with 15 cm × 75 µm C18 columns (5-μm particles with 100 Å pore size).

The MS/MS spectra were searched against the mouse RefSeq database (March 15, 2015) with SEQUEST in Proteome Discoverer (version 1.4, Thermo Fisher) using the same settings as previously described (21). The false discovery rate was set to 0.01 to eliminate identification of low-probability proteins. Missing values were replaced with 0.01 to be able to calculate ratios and *P* values. The mass spectrometry proteomics data have been deposited to the ProteomeXchange (https://www.ebi.ac.uk/pride/archive/) with the data set identifier PXD011790.

To confine the number of proteins for downstream analysis to those that best predict outcome, the R function stepAIC was used to build prediction models. For skin wound closure, proteins that correlated by Pearson’s correlation analysis with a minimum three of the five skin wound healing measurements (wound closure rate at *days 1*, *2*, or *3*, time to wound closure, or wound healing slope) were included in the initial model. To build a prediction model for end diastolic volume, proteins that correlated with minimum three out of four measurements for dilation (diastolic and systolic volume, and diastolic and systolic dimension) were included. For MI survival, the top 10 proteins that correlated with survival by Pearson’s correlation analysis (ranked by *P* value) were included. A combination of forward and backward selection was used to create the best fit model [lowest akaike information criterion (AIC)]. The best fit model was further reduced to the lowest number of variables without compromising prediction (adjusted *R*^2^).

### Validation of Selected Glycoproteins

Using the prediction models as a base, we selected five glycoproteins for validation: α_2_-macroglobulin (A2M), ELL-associated factor 1 (EAF1), apolipoprotein D (ApoD), vitamin D binding protein (VDB), and galectin 3 binding protein (Lgals3BP). A2M (NBP2-60631, Novus Biologicals), Lgals3BP (EKC41750, Biomatik), and VDB (DY4188-05, R&D Systems) were measured in plasma by enzyme-linked immunosorbent assay (ELISA) according to the manufacturer’s protocols. A2M (R&D Systems, Cat. No. AF5798, 1:1,000) in tissue and ApoD (Abcam, Cat. No. ab187513, 1:2,000) in plasma were measured by immunoblotting. Plasma (1 µL) or tissue (10 µg) was loaded on a 4–12% criterion Bis–Tris gel (Bio-Rad, Hercules, CA) and transferred to a nitrocellulose membrane (Bio-Rad). Recombinant ApoD (LSBio LS-G14539) was used as positive control and spleen as negative control for ApoD, whereas liver was used as a positive control and spleen as a negative control for A2M (34, 35). Membranes were stained with Pierce reversible protein stain (Thermo Scientific, Waltham, MA) to verify loading accuracy. For ApoD, the membranes were blocked with 5% nonfat milk (Bio-Rad) for 1 h at room temperature, incubated with primary antibody overnight at 4°C and secondary antibody (Vector Laboratories PI-1,000, 1:5,000) for 1 h at room temperature. For A2M, the iBind system (Invitrogen, Cat. No. SLF2020) was used according to manufacturer’s protocol. Chemiluminescence was detected using Amersham ECL Substrate (GE Healthcare, Waukesha, WI). Protein densitometry was quantified using iBright analysis software version 4.0.0 (Thermo Fisher Scientific). The protein densitometry for tissue samples was normalized to total protein stain. EAF1 (MyBiosource MBS2528345, 1:1,000) was measured in 2-µL plasma samples spotted directly on a nitrocellulose membrane using BioDot apparatus (Bio-Rad), following the same protocol as for ApoD immunoblotting. Kidney was used as a positive control and heart as a negative control for EAF1 (35).

### Human Evaluation

All participants gave written consent before participation in the study. The investigation conformed to the principles outlined in the Declaration of Helsinki. The human subject protocol was approved by the Institutional Review Board at the University of Mississippi Medical Center (IRB No. 2013-0164). Plasma was collected 48 h after admission in patients with MI (*n* = 41) or from healthy controls (*n* = 18). Patient characteristics are listed in Supplemental Table S1 (all Supplemental material is available at https://doi.org/10.6084/m9.figshare.17030042) (29).

The Human Glycosylation Antibody Array 1000 (RayBio, Cat. No. GAH-GCM-1000-4) contained preblocked glass slides coated with antibodies for 1,000 glycosylated proteins. The array was performed according to manufacturer’s instructions. The slides were incubated with human plasma (1:5 dilution) and washed to remove unbound proteins. Five unique biotin-labeled lectins were incubated with the array. Each lectin-bound respective glycan moieties on the captured proteins present on the glass surface. Streptavidin-conjugated fluorescent dye (Cy3 equivalent) was added to recognize the biotin attached to any bound lectin molecule. Chemidoc laser fluorescence scanning (Bio-Rad) was used to visualize the signals. Signals were quantified and normalized to reference spots. CXCL4 concentrations were previously reported from this dataset (29).

### Peritoneal Macrophage Stimulation

Mice were anesthetized with 2% isoflurane and ice-cold RPMI1640 medium (Life Technologies) containing 10% fetal bovine serum (FBS, Life Technologies) and 1% antibiotics (Life Technologies) was injected into the peritoneal cavity of the animals. Medium was removed from the peritoneal cavity and centrifuged at 250 *g* for 10 min. The unstimulated peritoneal macrophages were resuspended in RPMI1640 medium and seeded (10^6^ cells/well). After 2 h of incubation at 37°C, nonadherent cells were removed and the attached cells were stimulated with recombinant mouse ApoD (50 ng/mL, LSBio, Cat. No. G14539) for 24 h. After 24 h, the medium was removed, cells washed with PBS, and TRIzol Reagent (Invitrogen Life Technologies, Grand Island, NY) added to lyse the cells for RNA extraction.

For real-time RT-PCR analysis, RNA was extracted from isolated peritoneal macrophages using Purelink RNA (Invitrogen Life Technologies, Grand Island, NY) according to manufacturer’s protocol. RNA (500 ng) was reverse transcribed using RT2 First-Strand Kit (Qiagen, Valencia, CA). Gene expression of proinflammatory (M1) markers (*Ccl3*, *Ccl5*, *Il1b*, *Il6*, *Il12a*, *Tnfα*) and anti-inflammatory (M2) markers (*Arg1*, *Mrc1*, *Il10*, *Tgf-β1*, *Ym1*) were measured using Taqman gene expression assays (Life Technologies). Gene expression was calculated as 2^−ΔCt^ using hypoxanthine guanine phosphoribosyl transferase 1 (*Hprt1*) as the reference gene.

### Fibroblast Migration and Proliferation

Cardiac fibroblasts were isolated from naïve mice. The tissue was centrifuged at 250 *g* for 5 min, resuspended in DMEM/F12 medium (Gibco, Cat. No. 11320) with 1% antibiotic-antimycotic solution (Gibco, Cat. No. 15240) and 10% FBS (Gibco, Cat. No. 16000), and plated at 37°C and 5% CO_2_. Cells were used until *passage 3*. Primary skin fibroblasts were isolated from the biopsy punches as previously described (36).

Cardiac fibroblast proliferation was assessed using a colorimetric, 5-bromo-2-deoxyuridine (BrdU)-based assay as previously described (37, 38). BrdU was incorporated in newly synthesized DNA and quantified by measuring absorbance at 370 nm. Cells were incubated with apolipoprotein D (ApoD, 50 ng/mL, LS Bio, Cat. No. G14539) for 24 h before the BrdU assay.

Cardiac and skin fibroblast migration was assessed using electric cell-substrate impedance sensing (ECIS, Applied Biophysics) as previously described (39, 40). Cells were plated on gold film electrodes, and migration was measured as change in impedance as cells migrate and cover the electrode.

### Statistical Analysis

Data are reported as means ± SE. Two group comparisons of independent and dependent data were analyzed by unpaired and paired *t* test, respectively. Multiple group comparisons were analyzed by linear mixed effects model using a restricted maximum likelihood fit. Individual contrasts of least-squares means were adjusted using Tukey’s method. Pearson’s correlation analysis was used to determine correlation between two variables. Males and females were combined for statistical analysis, if not otherwise stated. All statistical analyses were performed in R version 4.0.4. A *P* value < 0.05 was considered significant.

## RESULTS

### Faster Skin Wound Closure Rates Predicted Survival after MI

Mice were given a 3-mm skin wound using a biopsy punch, and the wound was imaged daily until closure [Fig. 1*Ai*)]. Four weeks later, the same set of mice were given MI by permanent coronary artery ligation and followed for 7 days. Mice that survived until *day 7* were grouped as survivors (68%), and mice that died before *day 7* (32%) were grouped as nonsurvivors [Fig. 1*Aii*)]. Interestingly, survivors had faster skin wound closure compared with nonsurvivors (Fig. 1*B*). In addition, the skin wound closure rate, that is, 100% − (wound size/baseline wound size × 100), better predicted MI survival than commonly used cardiac functional variables (Fig. 1*C*), including infarct size (extent of myocyte necrosis), fractional shortening (cardiomyocyte contractility), end diastolic dimension (left ventricle dilation), and infarct wall thickness. Infarct size, measured at *day 7* in survivors and at autopsy in nonsurvivors, was not different between groups (Table 1). As expected, MI induced infarct wall thinning, reduced fractional shortening and ejection fraction, and increased systolic and diastolic dimensions and volumes (Fig. 1*D* and Table 1). These results indicate that the healing response to skin wounding was a robust predictor of MI cardiac wound healing.

**Table 1.** Echocardiography at baseline (day 0) or at MI day 3, and necropsy at MI day 7 or autopsy at the time of death did not differ between survivors and nonsurvivors

	*Day 0*		*MI Day 3*		*MI Day 7*		
	Survivors	Nonsurvivors	Survivors	Nonsurvivors	Survivors	Nonsurvivors	*P* Value
Heart rate, beats/min	468 ± 9	468 ± 9	475 ± 15	471 ± 19	492 ± 20	NA	0.99
Diastolic volume, µL	60 ± 2	60 ± 3	86 ± 3	98 ± 6	130 ± 6	NA	0.67
Systolic volume, µL	20 ± 1	20 ± 1	71 ± 3	83 ± 8	114 ± 5	NA	0.46
Stroke volume, µL	39 ± 1	39 ± 2	15 ± 1	15 ± 9	17 ± 1	NA	0.90
Ejection fraction, %	66 ± 0	66 ± 0	18 ± 1	16 ± 4	13 ± 1	NA	0.50
Infarct size, %	NA	NA	NA	NA	55 ± 1	60 ± 4	0.13
LV mass, mg	NA	NA	NA	NA	96 ± 5	102 ± 7	0.50
RV mass, mg	NA	NA	NA	NA	17 ± 1	19 ± 2	0.16
Lung mass wet, mg	NA	NA	NA	NA	253 ± 14	298 ± 26	0.10

Values are means ± SE. *P* value shows comparison between survivors and nonsurvivors using linear mixed effects model analysis for echocardiography and *t* test for necropsy. LV, left ventricular; MI, myocardial infarction; NA, not applicable; RV, right ventricular.

### Day 3 MI α_2_-Macroglobulin Linked to Day 3 MI Dilation and Predicted Future Survival

Because skin wound healing rates predicted MI wound healing, we hypothesized that common wound healing markers in plasma would reflect universal wound healing efficiency. Focusing on the extracellular matrix compartment, the plasma glycoproteome was mapped 3 days after skin-wounding (*D−25*) and at MI *D3* using mass spectrometry. A total of 1,299 glycoproteins were detected in MI *D3* plasma (Supplemental Data S1), of which 278 proteins were different between survivors and nonsurvivors (all higher in nonsurvivors; *P* < 0.05). The top 10 proteins ranked by *P* value were collagen α-1(III) chain, cytochrome P450 2C39, α_2_-macroglobulin (A2M), tyrosine-protein kinase Fer, multiple epidermal growth factor-like domains protein 8, coiled-coil domain-containing protein 180, ELL-associated factor 1 (EAF1), Lgals3BP, olfactory receptor 449, and MHC I like leukocyte 1. These 10 proteins were used to build a prediction model for MI survival using the R function stepAIC. The two proteins that best predicted survival (lowest AIC, highest adjusted *R*^2^) were A2M and EAF1 (Table 2). In combination, A2M and EAF1 were able to predict MI death in 66% of cases. Of the 278 glycoproteins that differed between survivors and nonsurvivors, proteins that correlated with a minimum three of four dilation measurements (diastolic and systolic volume and dimension) by Pearson’s correlation analysis were used to build a prediction model for *D3* and *D7* end diastolic volume. *D3* plasma C->U editing enzyme APOBEC2, E3 SUMO-protein ligase RanBP2, formin like protein 2, CD40 ligand, ankyrin repeat and SOCS box protein 17, and vomeronasal receptor Vmn2r106 in combination could explain 67% of the variability in *D3* end diastolic volume (Table 2). NudC-domain containing protein 3, kinesin like protein KIF3C, formin-like protein 2, V type proton ATPase subunit D, complement component C7, unconventional myosin Ia, ubiA prenyltransferase domain containing protein 1, and centrosomal protein of 131 kDa in combination could, on the other hand, explain 56% of the variation in *D7* end diastolic volume (Table 2).

**Table 2.** Plasma proteins in MI day 3 plasma that predict survival and day 7 dilation and tracked with day 3 dilation

	Zero-Order Correlation Coefficient (*r*)	β	SE	*P* Value	Adjusted *R*^2^
MI survival					
A2M	−0.70*	−6.81 × 10^−8^	3.22 × 10^−8^	0.034	Pseudo *R*^2^ 0.66
EAF1	−0.65*	−1.12 × 10^−8^	5.70 × 10^−9^	0.049	
*D3* diastolic volume					
APOBEC2	−0.60*	−2.23 × 10^−7^	4.88 × 10^−8^	<0.001	0.67
RanBP2	−0.49*	4.76 × 10^−9^	1.62 × 10^−9^	0.011	
FMNL2	−0.58*	−4.60 × 10^−8^	1.57 × 10^−8^	0.011	
CD40L	−0.44*	5.94 × 10^−8^	2.07 × 10^−8^	0.012	
ASB17	−0.46*	−2.14 × 10^−7^	8.46 × 10^−8^	0.024	
Vmn2r106	0.49*	1.23 × 10^−7^	6.73 × 10^−8^	0.089	
*D7* diastolic volume					
NUDCD3	−0.48*	−2.37 × 10^−6^	5.19 × 10^−7^	<0.001	0.56
KIF3C	−0.47*	7.18 × 10^−8^	1.95 × 10^−8^	0.0016	
FMNL2	−0.51*	−1.04 × 10^−7^	2.85 × 10^−8^	0.0017	
ATP6V0D1	−0.51*	1.22 × 10^−6^	4.11 × 10^−7^	0.0080	
C7	−0.43*	−2.21 × 10^−7^	7.52 × 10^−8^	0.0084	
MYO1A	−0.52*	−8.02 × 10^−7^	3.22 × 10^−7^	0.022	
UBIAD1	−0.39*	−1.29 × 10^−7^	5.45 × 10^−8^	0.029	
CEP131	−0.48*	5.47 × 10^−8^	3.13 × 10^−8^	0.096	

Proteins that best predict MI survival and dilation were selected using automatic model selection. Proteins are ranked by *P* value. *P < 0.05 for the zero-order correlation coefficient. A2M, α_2_-macroglobulin; ASB17, ankyrin repeat and SOCS box protein 17; APOBEC2, C->U editing enzyme APOBEC 2; ATP6V0D1, V type proton ATPase subunit d; C7, complement component C7; CD40L, CD40 ligand; CEP131, centrosomal protein of 131 kDa; *D3*, *day 3*; *D7*, *day 7*; EAF1, ELL-associated factor 1; FMNL2, formin-like protein 2; KIF3C, kinesin-like protein KIF3C; MYO1A, unconventional myosin Ia; NUDCD3, nudC-domain-containing protein 3; RanBP2, E3 SUMO-protein ligase RanBP2; UBIAD1, ubiA prenyltransferase domain-containing protein 1; VMN2R106, vomeronasal receptor Vmn2r106.

**Figure 2.**
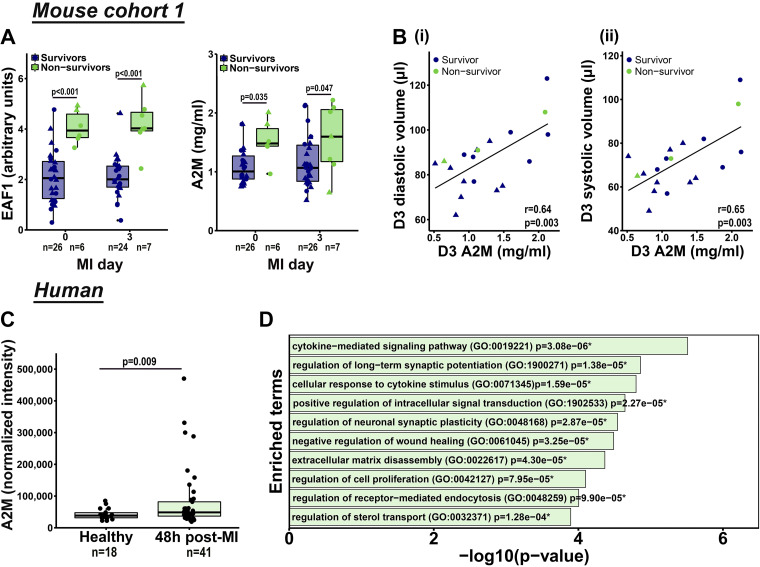
α_2_-Macroglobulin (A2M) correlated with dilation and predicted myocardial infarction (MI) death. ELL-associated factor 1 (EAF1) and A2M were higher in nonsurvivors both at baseline and MI *day 3* (*A*). Data were analyzed using linear mixed-effects model with Tukey’s post hoc test. Circles represent males and triangles represent females. Plasma A2M tracked with *D3* diastolic and systolic volume (*B*). Data were analyzed by Pearson’s correlation analysis. Circles represent males and triangles represent females. A2M increased in patients with MI (*C*). Data were analyzed by unequal variance *t* test. Gene ontology pathway analysis of proteins that correlate with A2M in humans (*P* < 0.05) revealed enrichment of the cytokine-mediated signaling pathway, cellular response to cytokine stimulus and negative regulation of wound healing, all biological processes involved in wound healing (*D*). *P < 0.05. *D3*, *day 3*. *n* represents number of animals (*A*) or subjects (*C*) in each group.

To validate the glycoproteome findings, we used a secondary method to measure plasma A2M by ELISA and EAF1 by immunoblotting. Higher levels of A2M and EAF1 were detected in plasma of nonsurvivors at baseline and at MI *D3* (Fig. 2*A*). A2M strongly correlated with diastolic and systolic volumes (Fig. 2*B*) and dimensions (Supplemental Fig. S1), which are strong predictors of progression to heart failure following MI (41, 42). In addition, A2M correlated with LV and lung weight at MI *D7* (Supplemental Fig. S1). The mice with higher A2M levels at MI *D3* had higher mortality. Dividing the group by the median of A2M plasma levels, mice above the median had sixfold higher mortality (38%) compared with mice below the median (6%, *P* = 0.005). EAF1 plasma levels did not correlate with any cardiac variable.

To investigate the clinical relevance of A2M, the plasma glycoproteome (1,000 glycoproteins) from patients with MI at 48 h after presentation (*n* = 41) and healthy controls (*n* = 18) was mapped using the RayBio Human Glycosylation 1000 Antibody Array. A2M increased in patients with MI compared with healthy controls (Fig. 2*C*). Of the 1,000 proteins analyzed (Supplemental Data S2), 50 proteins linked to A2M, with the strongest positive correlations being to forkhead box protein P3 (FoxP3), amyloid-β precursor protein (APP), and serum amyloid P-component (APCS; all *P* < 0.001, Supplemental Fig. S2). Fatty acid binding protein 3 (FABP3) was the only negative correlation (*P* = 0.043). Gene ontology pathway analysis of the 50 proteins revealed enrichment of the cytokine mediated signaling pathway, cellular response to cytokine stimulus, and negative regulation of wound healing as the top signaling pathways (Fig. 2*D*), supporting a strong connection between A2M and wound healing.

Collagen type III was the highest ranked protein by *P* value. At MI *D3*, there was fourfold higher collagen type III in the plasma of nonsurvivors after MI (*P* < 0.001, Supplemental Data S1). Collagen type III was not different in plasma after skin wounding (*D−25*, *P* = 0.31, Supplemental Data S3). The difference is likely due to dilution of the wounding proteins in the plasma, as the extent of collagen type III degraded or produced would be greater for the MI than the skin wound. An increase in degraded collagen type III in *D3* MI plasma would explain the increased LV rupture rate and mortality in nonsurvivors.

### A2M Is Protective during the Early Phase and Detrimental during the Late Phase of MI Wound Healing

**Figure 3.**
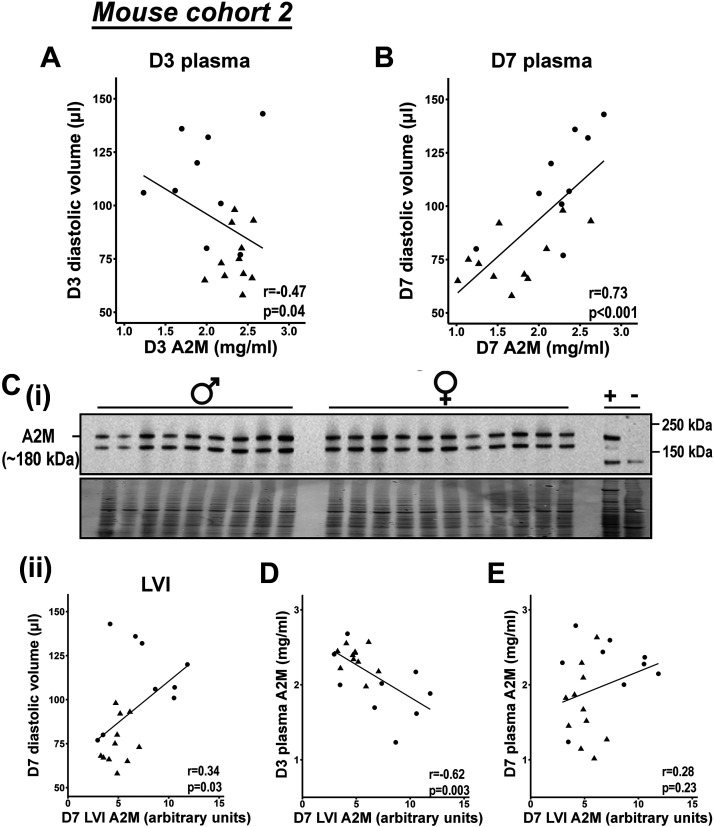
α_2_-Macroglobulin (A2M) showed a biphasic effect on myocardial infarction (MI)-induced dilation. In a second cohort of mice high levels of plasma A2M at MI *day* (*D*) *3* tracked with lower diastolic volume (*A*), whereas high levels of plasma A2M at MI *D7* tracked with higher diastolic volume (*B*). Similar to *D7* plasma, A2M in left ventricle infarct (LVI) tissue tracked with diastolic volume (*C*). Interestingly, A2M in *D3* plasma predicted A2M in tissue at *D7* (*D*), whereas A2M in *D7* plasma did not reflect tissue concentrations (*E*). All data were analyzed by Pearson’s correlation analysis. Circles represent males and triangles represent females.

A2M was further validated in a second cohort of mice, in which MI was induced without prior skin wounding. The survival rate in the first cohort was typical to what we and others have reported. (30, 43–45) The second cohort showed an exceptional survival rate (95%, *n* = 20/21) that linked to a slower dilation rate (Table 3, Supplemental Fig. S3) and plasma kinetics. The second cohort of mice at MI *D7*, therefore, was more similar to the first cohort at MI *D3*. Because the second cohort had a slower rate of remodeling progression, this revealed a biphasic U-shaped effect of A2M on dilation. Higher levels of plasma A2M at MI *D3* tracked with smaller end diastolic volume (Fig. 3*A*), whereas higher levels of plasma A2M at MI *D7* tracked with larger end diastolic volume (Fig. 3*B*). This indicates that A2M has a protective effect during the early phase of MI wound healing and is detrimental when it persists into the late phase. Similar to MI *D7* plasma concentrations, A2M in the left ventricle infarct (LVI) positively correlated with cardiac dilation (Fig. 3*C*). *D3* plasma A2M predicted *D7* LVI A2M (Fig. 3*D*), whereas *D7* plasma A2M did not reflect LV concentrations (Fig. 3*E*). This indicates that *D3* plasma A2M changes precede *D7* LVI A2M changes. These results validate that high levels of plasma A2M early on protect against MI-induced LV dilation.

**Table 3.** Echocardiography at baseline (day 0) and MI days 3 and 7, and necropsy at MI day 7 in the second cohort of mice

	*Day 0*	*MI Day 3*	*MI Day 7*
Heart rate, beats/min	482 ± 12	506 ± 9	520 ± 10
Diastolic volume, µL	54 ± 2	65 ± 2*	92 ± 6*#
Systolic volume, µL	20 ± 1	48 ± 2*	76 ± 6*#
Stroke volume, µL	34 ± 1	17 ± 1*	16 ± 1*
Ejection fraction, %	63 ± 2	26 ± 2*	19 ± 2*#
Infarct size, %	NA	NA	51 ± 2
LV mass, mg	NA	NA	91 ± 3
RV mass, mg	NA	NA	19 ± 0
Lung mass wet, mg	NA	NA	239 ± 17

Values are means ± SE. Data were analyzed using linear mixed effects model analysis and Tukey’s post hoc test. *P < 0.05 compared with *day 0*; #*P* < 0.05 compared with *day 3*. MI, myocardial infarction; NA, not applicable; LV, left ventricular; RV, right ventricular.

### ApoD Regulates Both Cardiac and Skin Wound Healing in Male but Not Female Mice

A total of 184 glycoproteins were detected in *D−25* plasma (Supplemental Data S3). To minimize the chance for false positives, only proteins that correlated with a minimum three of five skin wound healing measurement variables (wound closure rate at *days 1*, *2*, or *3*, time to wound closure, and wound healing slope), or three of four dilation measurements (diastolic and systolic volume and dimension) were selected for further analysis. Apolipoprotein D (ApoD) and vitamin D binding protein (VDB) met these criteria for skin wound healing. Both ApoD and VDB negatively correlated with skin wound healing rate (higher in slow healers), and together could explain 22% of the variance in skin wound healing at *D−25* (Table 4). In addition, ApoD also predicted later MI death (Table 4). The effect of ApoD on MI death was driven by males (males: *r* = −0.783, *P* < 0.001; females: *r* = −0.27, *P* = 0.25). The effect of ApoD on skin wound healing was evident in both males (*r* = −0.36, *P* = 0.09) and females (*r* = −0.50, *P* = 0.02), and the effect was combinatorial (*r* = −0.43, *P* = 0.004). There was no correlation between ApoD and LV dilation in either sex, separately or combined.

**Table 4.** ApoD, VDB, Lgals3BP, and CBG plasma concentrations at D−25 reflect current skin wound healing status and predict future MI survival and dilation

	Zero-Order Correlation Coefficient (*r*)	β	SE	*P* Value	Adjusted *R*^2^
*D3* wound closure rate
ApoD VDB	−0.43* −0.31*	−1.92 × 10^−7^ −3.88 × 10^−11^	6.52 × 10^−8^ 1.96 × 10^−11^	0.0053 0.054	0.22
MI survival					
ApoD	−0.50*	−4.26 × 10^−8^	1.49 × 10^−8^	0.0043	Pseudo *R*^2^ 0.22
*D7* diastolic volume					
Lgals3BP CBG	−0.58* 0.54*	−4.16 × 10^−8^ 4.82 × 10^−7^	1.34 × 10^−8^ 1.83 × 10^−7^	0.0046 0.014	0.44

Proteins that best predict wound closure rate, future myocardial infarction (MI) survival and dilation were selected using automatic model selection. Proteins are ranked by *P* value. *P < 0.05 for the zero-order correlation coefficient. ApoD, apolipoprotein D; CBG, corticosteroid binding globulin; *D3*, *day 3*; *D7*, *day 7*; *D−25*, *day −25*; Lgals3BP, galectin 3 binding protein; MI, myocardial infarction; VDB, vitamin D binding protein.

Six proteins predicted dilation at MI *D7* (corticosteroid binding globulin, discoidin domain containing receptor 2, Lgals3BP, G-protein coupled receptor 158, serine protease inhibitor A3K, and tRNA selenocysteine 1 associated protein 1). Of these, Lgals3BP and corticosteroid binding globulin (CBG) were identified by automatic model selection to be the best predictors of dilation. Higher levels of Lgals3BP tracked with lower end diastolic volume, indicating a protective effect of Lgals3BP on dilation. The opposite effect was seen for CBG. In combination, Lgals3BP and CBG could explain 44% of the variation in diastolic volume at *D7* (Table 4).

**Figure 4.**
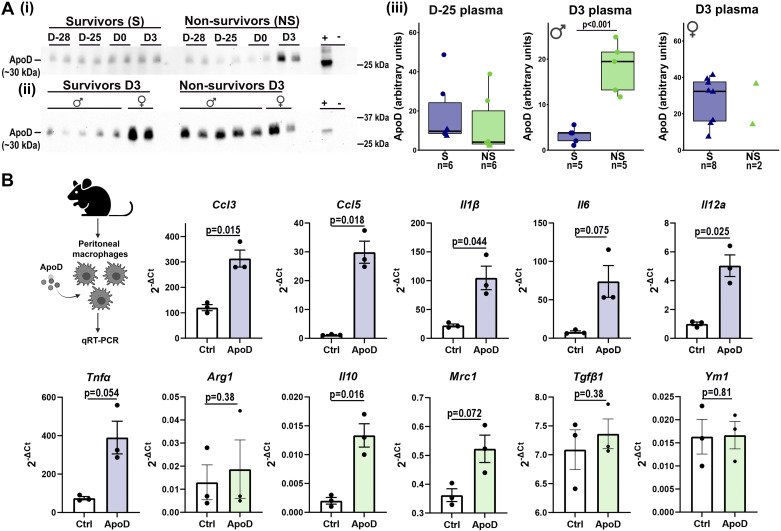
Apolipoprotein D (ApoD) stimulated macrophages to promote inflammation. ApoD was higher in plasma of nonsurvivor male mice at myocardial infarction (MI) *day 3* (*A*). *D−25* plasma ApoD data (*iii*) were derived from three different blots run under same conditions. The two other blots are shown in Supplemental Fig. S6. Data were analyzed by unpaired *t* test. ApoD stimulated expression of proinflammatory genes (*Ccl3*, *Ccl5*, *Il1β*, *Il12a*) in peritoneal macrophages isolated from male mice (*B*). Data were analyzed by paired *t* test. Image showing experimental design was created with BioRender and published with permission (accessed on 5 January 2022) (26). Arg1, anti-inflammatory arginase 1; Ccl3, C-C motif chemokine ligand 3; Ccl5, C-C motif chemokine ligand 5; Il1β, interleukin-1β; Il6, interleukin 6; Il10, interleukin 10; Il12a, interleukin 12a; Mrc1, mannose receptor C-type 1; Tnfα, tumor necrosis factor-α; Tgfβ1, transforming growth factor-β1; Ym1, chitinase-like protein 3. *D−25*, *day −25*. *n* represents number of animals in each group.

ApoD, VDP, and Lgals3BP were selected for validation. By immunoblotting, ApoD was not different between survivors and nonsurvivors at *D−25* indicating glycosylation of ApoD instead of protein levels was the main change during skin wound healing (Fig. 4*A*). ApoD was higher in plasma of nonsurvivor male mice 3 days after MI (Fig. 4*A*). In females, MI *D3* plasma ApoD levels were high in both survivors and nonsurvivors. Sex differences in expression regulation is partially explained by the presence of estrogen response elements in the ApoD promoter (46). In patients, ApoD did not change at 48 h after presentation of MI, and a longer follow-up period may be necessary to compare survivors with nonsurvivors. VDB and Lgals3BP showed no differences by ELISA between survivor and nonsurvivor mice at *D−25* or *D3*, indicating glycosylation of the protein was the index change (Supplemental Fig. S4). Overall, these results revealed that ApoD regulates both skin and cardiac wound healing in male mice.

### ApoD Induces Macrophages to Adopt a Proinflammatory Phenotype

Excessive inflammation and reduced fibroblast proliferation delays both skin and cardiac wound healing (6, 10, 47). In peritoneal macrophages from male mice stimulated with ApoD (50 ng/mL), multiple proinflammatory genes (*Ccl3*, *Ccl5*, *Il1β*, and *Il12a*) were elevated (Fig. 4*B*). Only one anti-inflammatory gene (*Il10*) was increased out of five evaluated (*Mrc1*, *Arg1*, *Tgfβ1*, and *Ym1)*. ApoD did not stimulate skin or cardiac fibroblast proliferation or migration (Supplemental Fig. S5), indicating the main effect of ApoD was to promote inflammation in the macrophages.

## DISCUSSION

The goal of this study was to determine if skin wound healing patterns could predict future responses to MI. A major finding was that skin wound closure rate predicted MI death. Fast skin wound healers had more efficient cardiac healing after MI, a finding supported by the higher MI survival rates in fast skin wound healers compared with slow skin wound healers. Following MI, two plasma proteins, EAF1 and A2M, predicted MI death in 66% of cases. In addition, A2M strongly correlated with early and late dilation, and dilation is already a well-known predictor of future heart failure development and death. We found that ApoD regulated both skin and cardiac wound healing in male mice by promoting inflammation. Combined, our results reveal that the skin is a mirror to the heart and that common pathways link wound healing across organs.

Wound healing across organs shares remarkable similarities. Tissue injury generically induces an inflammatory response followed by inflammation resolution, tissue repair, and scar formation (11). In this study, we showed that not only do they share similar cellular and molecular processes, tissue repair efficiency in one organ can predict tissue repair efficiency in other organs. Thus, skin wound healing may be used to screen for patients that are at higher risk of developing heart failure after an MI. We have previously shown that extent of dilation does not predict MI survival (48), and skin wound healing predicted MI death better than the cardiac indices infarct size, contractility, dilation, and wall thinning. In humans, infarct size approaching 20% would be lethal; mice, on the other hand, can survive at least 6 mo with infarct sizes of ∼50% (44, 49–51). In mice, the majority of MI deaths occur between *days 3* and *7* (30, 44). As our results showed, there was no correlation between infarct size and survival for infarct sizes in the 35–60% range.

A2M was a strong predictor of MI death and correlated with LV dilation in two different cohorts of mice. The absence of skin wounding in the second cohort of animals in combination with comparable results in both cohorts indicate that skin wounding had no preconditioning effect on the MI response. A2M is a broad spectrum protease inhibitor and an acute phase protein involved in nonspecific inflammatory response to tissue injury (52). Interleukins and tumor necrosis factor-α released during tissue injury stimulate hepatocytes to increase production of A2M (53). In accordance with our data, Haines et al. (54) showed that patients who died within 1 yr of MI had higher levels of A2M at hospital admission compared with patients who survived. The increased A2M in our patient cohort indicates that A2M is involved in human infarct healing. A more extensive analysis and longer follow-up of patients with MI is warranted, in particular giving the U-shaped response observed in the mouse cohorts. As with the inflammatory response during MI wound healing (45, 55, 56), A2M is not only detrimental, high levels seem to be beneficial during the early phase of wound healing. A2M may protect early by limiting proinflammatory cytokine activity, by inhibiting proteases to protect from rupture, and by stimulating macrophage phagocytosis (57–62). A2M may exacerbate later after MI by binding and inactivating TGFβ to prevent scar formation or reduce scar quality (63–65). In addition to the acute effects during tissue injury, A2M has direct effects on the heart. Tail vein injection of A2M induces cardiac hypertrophy in rats and direct stimulation of ventricular myocytes induces hypertrophic cell growth through the ERK1,2 and PI3-kinase/Akt pathways (58, 66).

EAF1 was another strong predictor of MI death. EAF1 is a positive regulator of the elongation factor ELL which in turn stimulates overall transcription rate by RNA polymerase II (67). EAF1 did not correlate with any of the cardiac physiology variables indicating that its main effect is on wound healing mechanisms independent of organ physiology. Thus, EAF1 is a biomarker of generic poor wound healing.

We found that ApoD regulates both skin and cardiac wound healing in male mice. ApoD is expressed in many tissues and shows diverse glycosylation profiles specific to the expression site (68). High levels have been linked to inflammatory neurological disorders such as Parkinson’s disease, Alzheimer’s disease, and multiple sclerosis (68–70). During the acute phase of MI, ApoD is known to be released from damaged myocytes (71). Here we showed that ApoD regulates inflammation by directly stimulating macrophages to adapt an inflammatory phenotype. Proinflammatory macrophages are key regulators of tissue repair across organs (11). In addition, ApoD was a strong predictor of survival in male mice following MI. ApoD may be used as a biomarker for risk stratification in males; however, further studies evaluating the role of ApoD in MI are warranted.

In conclusion, wound healing across organs shares common pathways. Tissue repair efficiency in one organ can predict tissue repair efficiency in a different organ. We showed that faster skin wound healing rate is a predictor of more favorable outcome after MI, and skin wound healing may be used to screen for patients that are at risk of developing heart failure.

## DATA AVAILABILITY

Mass spectrometry data have been deposited to the ProteomeXchange (https://www.ebi.ac.uk/pride/archive/) with dataset identifier PXD011790.

## SUPPLEMENTAL DATA

10.6084/m9.figshare.17030042 Supplemental Figs. S1–S5, Supplemental Table S1, and Supplemental Data S1–S3: https://doi.org/10.6084/m9.figshare.17030042.

## GRANTS

This work was supported by National Institutes of Health Grants U54GM115458 (to M.L.L.) and HL137319 (to M.L.L.), Biomedical Laboratory Research and Development Service of the Veterans Affairs Office of Research and Development Grant 5I01BX000505 (to M.L.L.), and Swedish Society for Medical Research Grant P19-0144 (to M.B-A.).

## DISCLOSURES

Dr. Lindsey is the editor in chief of the *American Journal of Physiology-Heart and Circulatory Physiology* and was not involved and did not have access to information regarding the peer-review process or final disposition of this article. An alternate editor oversaw the peer-review and decision-making process for this article. No conflicts of interest, financial or otherwise, are declared by the authors.

## AUTHOR CONTRIBUTIONS

M.J., M.B-A., and M.L.L. conceived and designed research, performed experiments, analyzed and interpreted data, prepared figures, and drafted manuscript. All authors edited and revised manuscript and approved final version.
